# Design and validation of a novel low-cost open paediatric inguinal herniotomy simulator

**DOI:** 10.1007/s00383-026-06486-4

**Published:** 2026-06-20

**Authors:** Tanay Bapna, Samuel J. A. Robinson, Damir Ljuhar, Maurizio Pacilli, Ramesh M. Nataraja

**Affiliations:** 1 Department of Paediatric Surgery & Monash Children’s Simulation, Monash Children’s Hospital, 246 Clayton Road, Clayton, Melbourne, 3168 Australia; 2 Departments of Paediatrics & Surgery, School of Clinical Sciences, Faculty of Medicine, Nursing and Health Sciences, Monash University, Melbourne, Australia

**Keywords:** Simulation, Surgical simulator, Paediatric surgery, Simulation-based education

## Abstract

**Background:**

Simulation-based education is a valuable tool in paediatric surgery. Inguinal hernias are a common paediatric surgical presentation with an incidence of 1–5% in full-term infants. While previous simulators have been created for adult inguinal hernias and laparoscopic paediatric inguinal hernias, there are limited models for the open paediatric procedure. We aimed to create and validate a simulator for a paediatric open inguinal hernia repair.

**Methods:**

Essential procedural steps of an open inguinal herniotomy were determined, and a low-cost paediatric simulator was developed. Novices (medical students) and experts (paediatric surgeons) were recruited. A questionnaire on an 11-point Likert-type scale assessed face validity, content validity, and functional task alignment. Median scores > 7.0/10.0 were deemed valid. The total number and type of procedural errors were assessed to determine construct validity. Statistical analysis included Fisher’s exact test, a p-value < 0.05 was considered significant.

**Results:**

A total of 34 participants were recruited (expert:10, novice:24). The simulator received favourable median scores in the following content validity domains: useful for surgical trainees (7.0/10.0), useful to train open surgical skills (7.5/10.0), and logical flow of procedural steps (9.0/10.0). It was not deemed a valuable training tool for experts (2.0/10.0). The simulator had median scores below 7.0/10.0 for face validity domains, including its tissue and visual realism. Construct validity was demonstrated by novices making more errors on the simulator, *p* = 0.02.

**Conclusions:**

This low-cost open inguinal herniotomy simulator demonstrated content validity, construct validity, and a high degree of functional task alignment.

**Supplementary Information:**

The online version contains supplementary material available at 10.1007/s00383-026-06486-4.

## Introduction

Simulation-based education (SBE) has become integral to surgical training, particularly in the context of reduced operative time [1–4]. SBE allows for the development of surgical skills in a safe learning environment and adapts to learners’ diverse training requirements through the use of surgical simulators [5].

The development of effective surgical simulators typically follows a structured framework: identifying specific training needs, designing the educational program, and specifying the simulation medium before conducting formal validation [6–9] The validation of the simulator follows this, which is typically conducted through experimental studies comparing novice and expert performance. Common terms in simulator validation include functional task alignment (resemblance to clinical operative environments), face validity (visual and tissue realism), content validity (utility as a training tool), and construct validity (ability to distinguish skill levels) [10–12]. Currently, there are no well-established guidelines as to a specific numerical benchmark that deems a simulator to be validated [6].

In paediatric surgery, the importance of simulation is especially highlighted with the technical complexity of cases, small operative area, and potentially limited exposure to cases [13]. Paediatric inguinal hernias remain a common surgical presentation with an incidence of 1–5% in full-term babies [14]. While multiple validated laparoscopic paediatric inguinal herniotomy simulators are available, there are limited published validated simulators for the open surgical technique [13, 15]. We aimed to design and validate a low-cost open paediatric herniotomy model targeted at surgical trainees on functional task alignment, face, content, and construct validity.

## Methods

### Simulator development

The design of this study was based on the conceptual framework proposed by Ericsson et al. that highlights the importance of repeated deliberate practice with immediate feedback to achieve competency in a task [16].

**Fig. 1:**
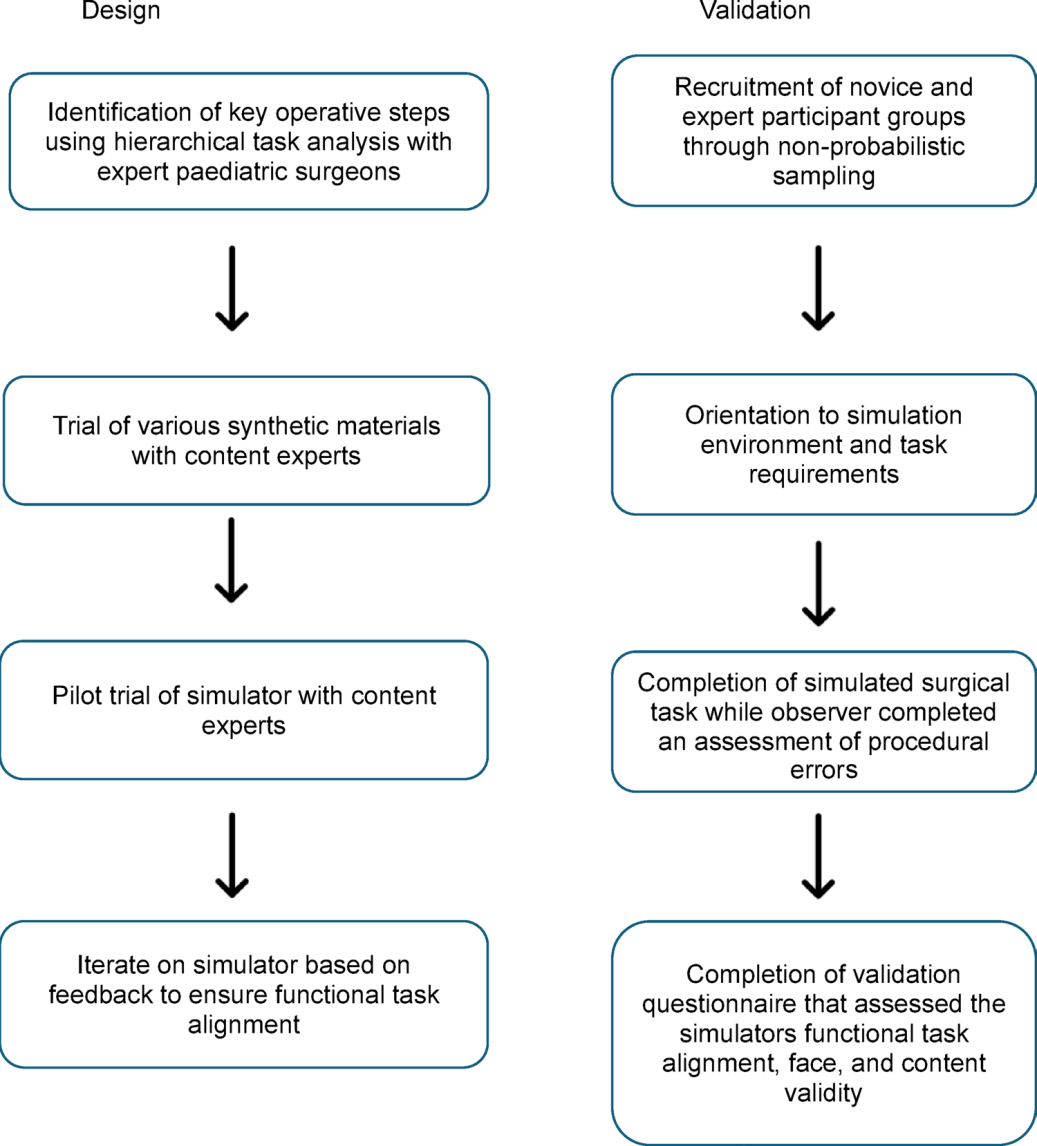
Design and validation framework for the development of the simulated inguinal hernia trainer.

The key clinical and operative steps involved in performing an open paediatric inguinal herniotomy were identified through a hierarchical task analysis with an expert panel utilising rapid cycle feedback [17]. The specific steps critical to gain competency were then chosen to be represented in the simulator through further discussions with expert paediatric surgeons. Eight out of the ten clinical operative steps were represented in the trainer. Various synthetic materials were tested to represent the abdominal wall and inguinal canal layers accurately. The simulator underwent several trial versions in which various expert paediatric surgeons provided feedback on its visual realism and functional task alignment. These experts were subsequently excluded from the validity assessment of the simulator to prevent bias. With the creation of the third version of the simulator using different materials based on this feedback, further validation was performed by a group of six independent consultant paediatric surgeons. In this trial, the polyethylene plastic material selected as Scarpa’s fascia was unable to hold sutures. Therefore, this was selected to be excluded as an operative simulated step on the simulator. Utilising this process of refinement simulator testing, the fourth and final simulator of the model was then developed and used during participant recruitment. The design and validation framework utilised is demonstrated in Fig. 1.

**Fig. 2:**
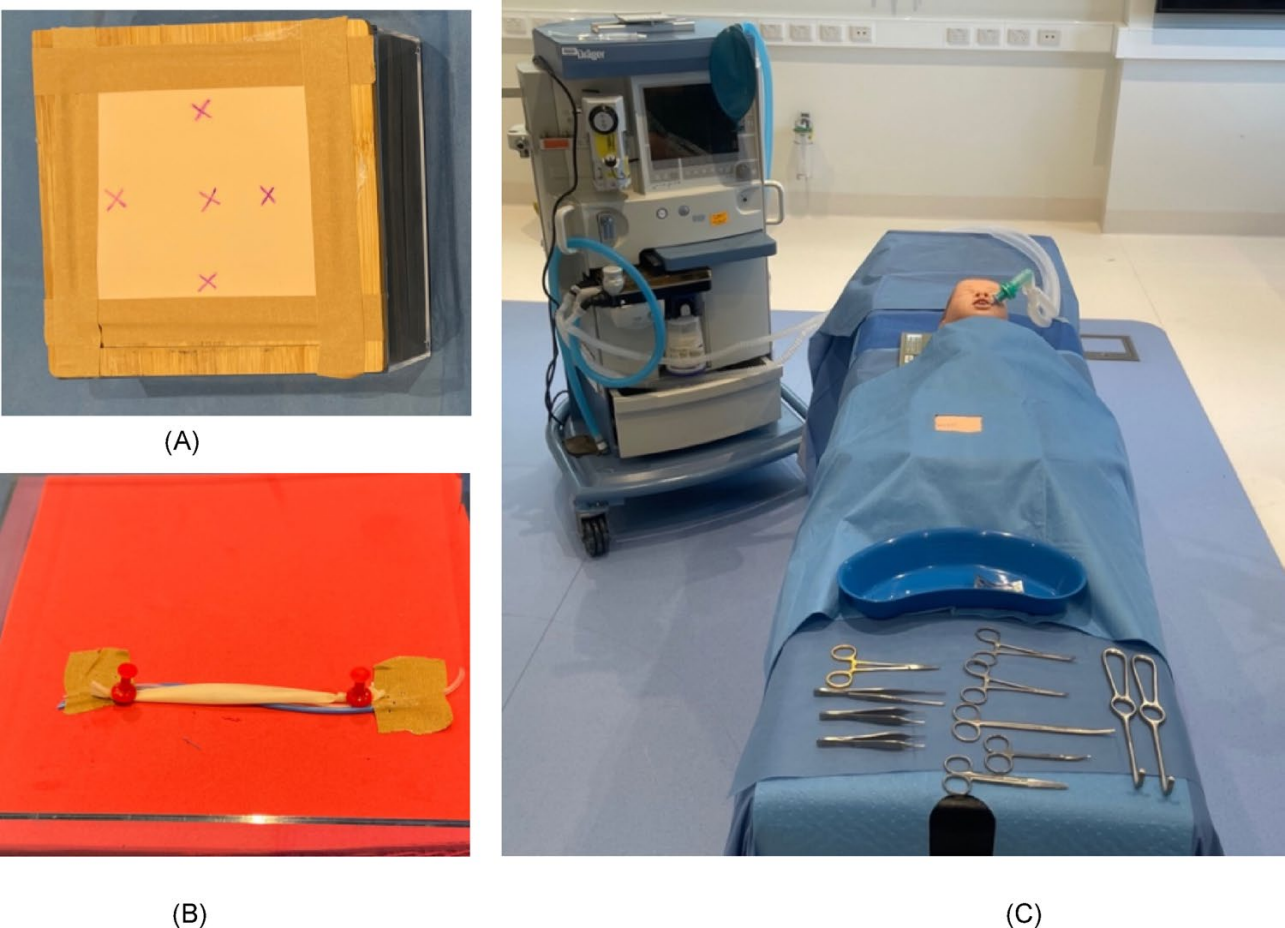
Complete simulated inguinal hernia model, **a** Simulated inguinal hernia box trainer, **b** Hernia sac with Vas deferens (transparent tubing) and vessels (blue tubing), **c** Final complete simulation set-up

Table 1 displays the materials used to construct each layer of the simulator and the corresponding cost. Each abdominal insert was used four times before being disposed of, with the location for each optimum skin incision demarcated by an “X” on the model to compensate for the absence of normal anatomical landmarks. A full-term infant torso simulator and a surgical drape were also placed over the box simulator to improve the visual realism of the simulation activity to promote participant engagement and immersion within the simulation. Additional assembly instructions are included in Supplementary Information. The approximate cost for each abdominal insert was AUD$5 with a per-use cost of AUD$1.30. The box-trainer is shown in Fig. 2a, and the full set-up is displayed in Fig. 2c.

**Table 1:** Comparison of procedural steps between a clinical and simulated paediatric open inguinal herniotomy

Steps of a clinical paediatric open inguinal herniotomy	Steps of the simulated paediatric open inguinal herniotomy
A 1–2 cm skin incision lateral to the pubic tubercle	A 1–2 cm skin incision
Dissection of Scarpa’s fascia	Dissection of Scarpa’s fascia
Dissection of the external oblique	Dissection of the external oblique
Identification of external ring of the inguinal canal	N/A
Identification of the hernia sac	Identification of the hernia sac
Separation of vessels and vas deferens from hernia sac	Separation of vessels and vas deferens from hernia sac
Division and ligation of hernia sac	Division and ligation of hernia sac
Closure of external oblique	Closure of external oblique
Closure of Scarpa’s fascia	N/A
Closure of skin incision	Closure of skin incision

### Experimental design

Participants were recruited via non-probabilistic snowball sampling. We recruited medical students as a novice group and senior paediatric surgical registrars or consultants as an expert group. Senior paediatric surgical registrars were defined as those at postgraduate year five or greater. Third or fourth-year medical students who could instrument-tie simple interrupted sutures were eligible to participate. Only expert performance and feedback were used for the final validation process of the simulator. The novice grades were used in the assessment of the content validity.

Each participant was shown an instructional video of an expert surgeon completing the simulated task. Additionally, participants were provided with a standardised step-by-step procedural guide, a diagram with the labelled layers of the model, and a labelled diagram of all the equipment available. Before starting the simulated task, all participants performed a warm-up exercise of one and three simple interrupted sutures on synthetic neoprene material. A designated surgical assistant (either SR or TB) was available during all trials to assist with retraction but did not provide any further assistance with any other aspects of the procedure. Participants were given 15 min to complete the task. While participants completed the task, a research team member completed a procedural checklist to assess whether the steps of the procedure were followed and if any of the anatomy was damaged during the task. Errors were documented in a binary “Yes or No” format and were defined as damage to an unintended structure while completing the task. There was also an opportunity to document errors using a free textbox. The procedural checklist can be found in Supplementary Information.

### Validation questionnaire

The validation questionnaire assessed the model’s functional task alignment, face, and content validity. The questionnaire was only completed by the expert group of paediatric surgeons using the online survey platform Qualtrics™ (Qualtrics, Provo, UT). The questionnaire consisted of three functional task alignment statements, seven face validity statements and four content validity statements that were developed with expert simulation and surgical input. Participants rated the statements on a 11-point Likert-type scale, with 0 being completely inaccurate and 10 being completely accurate. In line with other similar studies, the simulator was considered to be validated in a particular domain if the median expert rating was greater than 7/10 [6]. Participants also had the opportunity to provide free-text feedback about the simulator.

### Statistical analysis

Data was analysed using GraphPad Prism™ 9.5.1 (GraphPad Software, Boston, Massachusetts, USA). Continuous data was summarised using descriptive statistics such as mean, median, standard deviation (SD), and interquartile range. Parametric inferential tests, such as unpaired t-tests, were used for normally distributed data. Categorical data was analysed using Fischer’s exact test. A p-value of less than 0.05 was considered statistically significant.

## Results

A total of 34 participants completed the simulation, 24 novices and 10 experts. All expert participants had performed more than 100 open surgical procedures as the primary operator compared to the novice group, who had performed 0 open surgical procedures.

### Validation of the simulator

#### Face validity, realism and operative steps

**Fig. 3:**
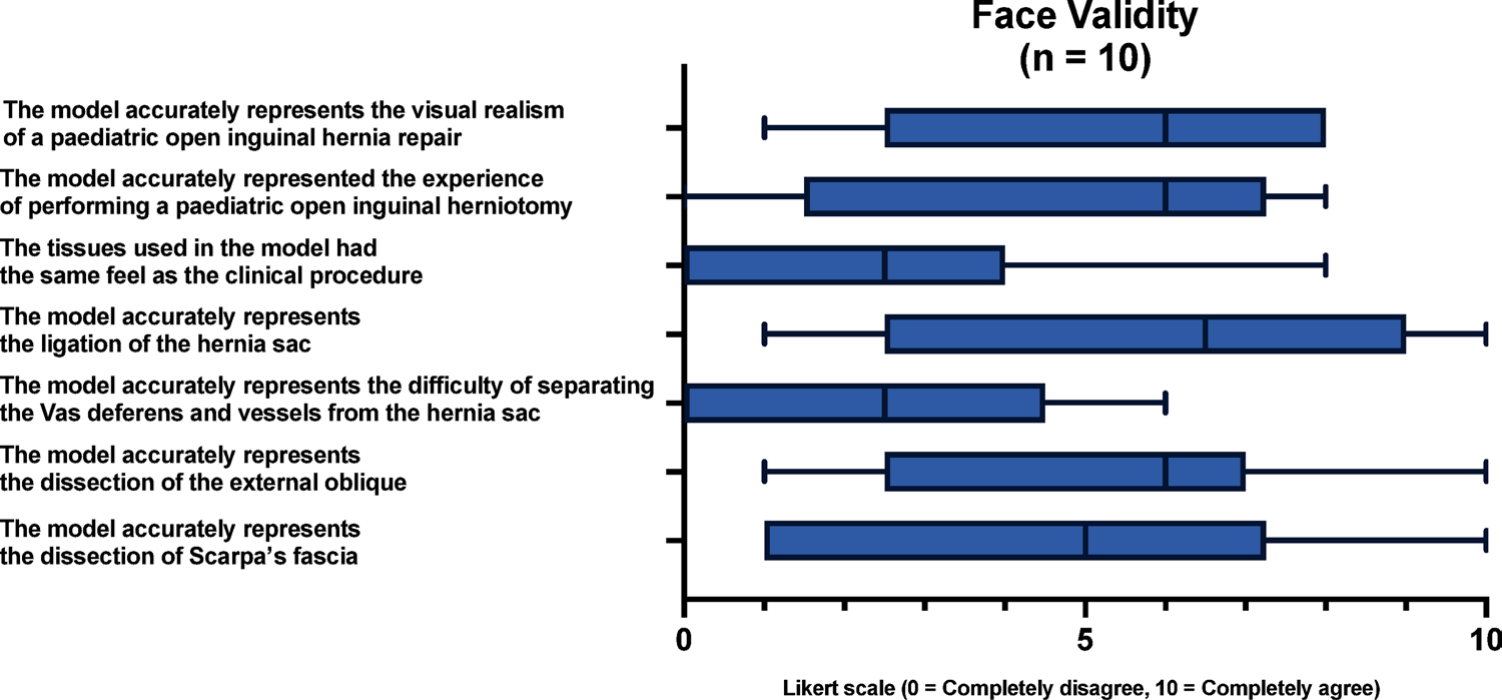
Face validity domains assessed by expert participants on the open paediatric inguinal herniotomy simulator

Face validity was assessed by the expert group’s rating of the visual and tissue realism of the simulator (*n* = 10). The model’s overall visual realism had a moderate score of 6.0/10.0 (IQR 2.5–8.0). The recreation of the experience of performing the clinical procedure had a score of 6.0/10.0 (IQR 1.5–8.0). The model’s tissue realism and separation of the Vas deferens and vessels received a score of 2.5/10.0 (IQR 2.5–8.0) and 2.5/10 (IQR 0.0–6.0), respectively. The accuracy in the representation of the ligation of the hernia sac was 6.5/10.0 (IQR 2.5- 9.0). The dissection of the external oblique and Scarpa’s fascia were rated 6.0/10.0 (IQR 2.5–7.0) and 5.0/10.0 (IQR 1.0–7.3), respectively. Within each domain, a wide range of scores was noted. Figure 3 displays the face validity outcomes on a box and whisker plot.

#### Training potential

**Fig. 4:**
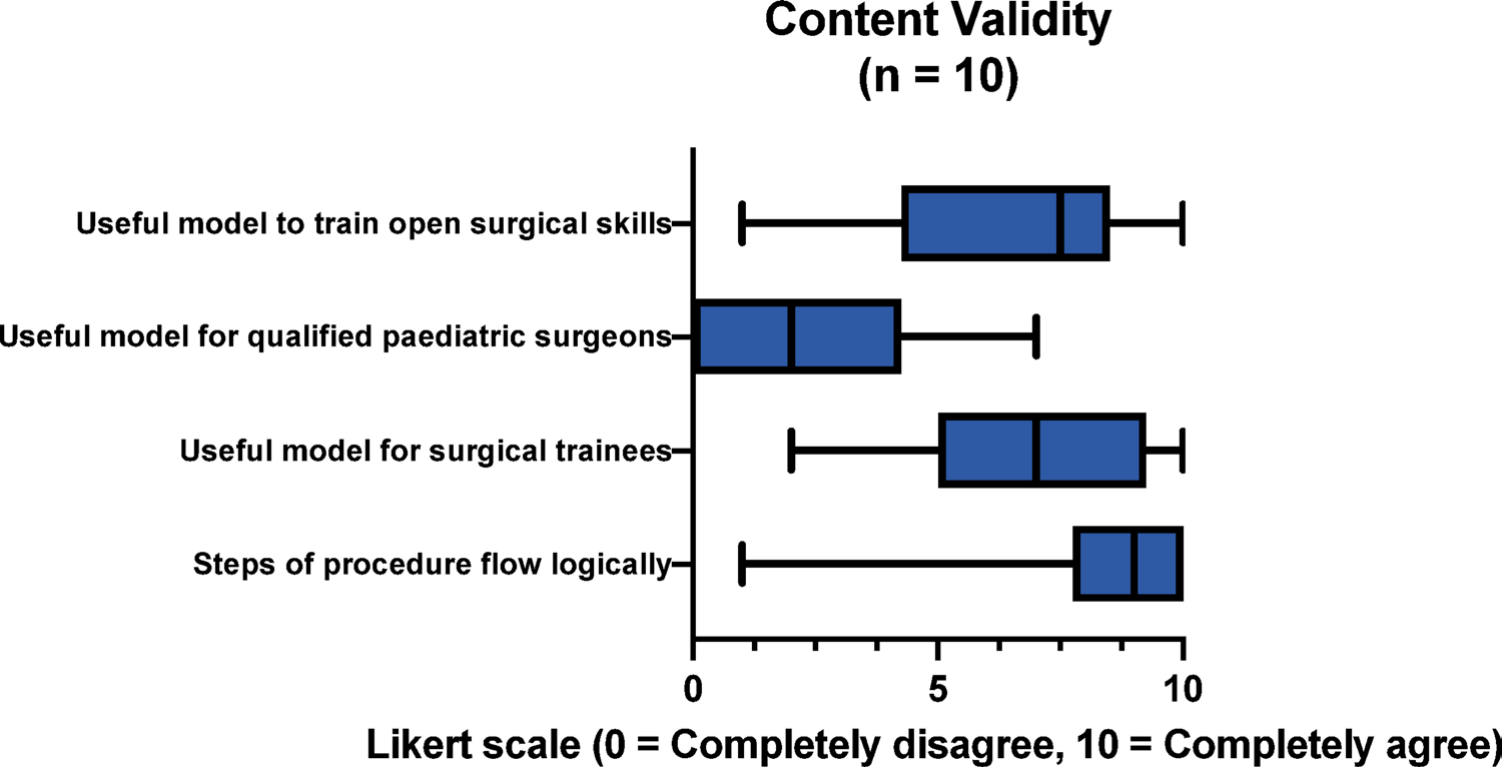
Content validity domains assessed by expert participants on the simulated open paediatric inguinal herniotomy

The simulator was rated highly by experts on its representation of the procedural steps with a median score of 9.0/10.0 (IQR 7.8–10.0). Most participants also found that the simulator would be useful as a training tool for surgical trainees and to train general open surgical skills with a median rating of 7.0/10.0 (IQR 5.0–9.3) and 7.5/10.0 (IQR 4.3–8.5), respectively. However, it was seen to be less beneficial for qualified paediatric surgeons as expected, with participants rating it 2.0/10.0 (IQR 0.0–4.3). Figure 4 displays the content validity outcomes on a box and whisker plot.

### Content validity

Content validity was assessed with the amount of critical procedural errors. In the novice group, 14 total errors were made compared to 1 in the expert group, *p* = 0.02. In the novice group, there was a much higher damage rate to either the Vas deferens or the vessels 8/24 (33.3%), compared to 0/10 (0%) in the expert group, *p* = 0.07.

## Discussion

The described simulated inguinal hernia simulator demonstrated both content validity, construct validity, and excellent functional task alignment [11]. Experts agreed that the model accurately recreated the procedure steps and would be helpful for surgical trainees and in training open surgical skills. The model was rated relatively low in its utility as a training tool for qualified paediatric surgeons as expected. Consultant paediatric surgeons have already achieved competency in the task and were unlikely to gain further benefit from the low cost simulation.

The content validity outcomes indicate that the model would allow a naïve learner to understand the basic steps of the procedure and improve their general open surgical skills.

Construct validity was assessed by the total number of errors participants made while completing the simulated procedure, which was statistically different. This identifies the simulators’ ability to differentiate between novice and expert performance. While damage to the vas deferens or vessels was more common in the novice group (33.3% vs. 0%), this was not statistically significant. The functional task alignment of the model was excellent as it was determined that the steps of the procedure flowed logically and had excellent fidelity. This identifies that the steps involved in completing the simulated task were similar to the clinical operations.

The simulator received relatively low scores on face validity domains, including both visual and tissue realism, which was expected given the low-cost materials. We attempted to maintain a level of visual realism by using an intubated infant torso in a simulated operating theatre environment with a surgical assistant. The materials used in the simulator allowed for the psychomotor aspect of the task to be maintained, even with reduced tissue realism. It has also been well-established in simulation that limited physical resemblance does not necessarily reduce the educational value of a simulator as per the work of Hamstra and colleagues [11]. While the key operative step of separating the Vas deferens and vessels was not highly rated, the general aspects of the model allow it to remain a useful tool in providing junior trainees with experience in the approach to an open inguinal herniotomy. This is key consideration as a simulator might not include all the clinical steps but the skills acquired for the majority of them decrease the cognitive overload of the junior surgeon in the operation. Given the similarities in the approach between open inguinal herniotomies, orchidopexy, and hydrocele, the simulator can teach trainees the transferable operative steps, including fascial dissection, exposure, identifying relevant structures, fascial repair, and skin suturing.

While the simulator had value for surgical trainees, it had some limitations. We demonstrated the ability of the simulator to differentiate between novice and expert performance using the total number of errors made. To further improve the functional task alignment, participants may wear sterile gloves to increase the resemblance to the operative environment. Additionally, wide interquartile ranges (IQR) were demonstrated across multiple domains, indicating some variability in the opinion of the expert respondents. The variation may be attributed to a lack of familiarity with low-visual fidelity surgical models for surgical skill acquisition, leading to lower ratings. We acknowledge the poor rating on a key operative step of the separation of the Vas deferens and vessels as a key limitation of the model. We believe the utility in the model as a surgical training for various open surgical procedures remains.

Most available open inguinal herniorrhaphy simulators train the Lichenstein technique, which is performed in adult operations rather than the paediatric herniotomy technique [18–21]. The validated paediatric inguinal hernia repair models train the laparoscopic approach [22–24]. Recent work by Heo et al. and Malik et al. described the development and validation of a 3D-printed paediatric inguinal hernia simulator simulating both open and laparoscopic repair techniques [25, 26]. These models demonstrated face, content, and construct validity and provided broader procedural versatility, including simulation across male and female anatomical models. The reported initial production cost of the 3D-printed simulator exceeded AUD$450, compared with approximately AUD$5 for the abdominal insert used in this study. Although the subsequent per-use costs of both models were comparable AUD$1.82 for the 3D printed model versus AUD $1.30 in this study. Compared with these 3D-printed simulators, our model was designed with a greater emphasis on accessibility and low-cost reproducibility using widely available materials. While the 3D printed model provided greater visual realism, it required access to 3D-printing infrastructure and more complex model construction. Both approaches demonstrate validity and utility in paediatric surgical simulation.

The open paediatric inguinal herniotomy simulator in this study developed may allow trainees to achieve an appropriate level of proficiency through deliberate practice and provision of constructive feedback, before the transition to the operating theatre. This may increase patient safety and give the trainee more confidence in their transition towards operative independence. This simulator is particularly relevant in a lower-resourced setting due to its low-cost nature [27]. The low cost, low technology, and accessibility of this simulator make it ideal for such environments. There is potential for this simulator to be used as part of paediatric surgical educational programs in various settings to improve surgical trainees’ skill acquisition across a variety of surgical operations where a similar operative approach is taken, including orchidopexies and hydrocele repairs.

## Conclusion

We have developed and validated a low-cost open paediatric inguinal herniotomy simulator. The simulator demonstrated content and construct validity with its utility as a training tool and its ability to differentiate between novice and expert performance. This simulator has a variety of potential further applications, including in both low and high-resourced settings, to assist in training surgeons in their skill development.

## Supplementary Information

Below is the link to the electronic supplementary material.

Supplementary Material 1

## Data Availability

No datasets were generated or analysed during the current study.
