# Supplementary material for: Design and validation of a novel low-cost open paediatric inguinal herniotomy simulator
Source: Pediatr Surg Int. 2026 Jun 20;42(1):264. doi: 10.1007/s00383-026-06486-4 (PMC13283189; doi:10.1007/s00383-026-06486-4)
Supplement: Supplementary file 1 — Supplementary Material 1 [file 383_2026_6486_MOESM1_ESM.docx]

**Supplementary Materials - Index**

| **Supplementary Methods** |  |
| --- | --- |
| Detailed instructions to construct the open paediatric inguinal hernia model. | *2* |
| Steps to construct box and attachment of hernia sac, Vas deferens, and vessels | *2* |
|  |  |

**Supplementary Methods**

**Detailed instructions to construct the open paediatric inguinal hernia model.**

*Steps to construct abdominal wall inserts.*

1. Gather all equipment including artificial skin, polyethylene plastic, brown spandex cloth, white latex balloon, and IV tubing and blue vessel loops.
2. Using scissors, cut a 12.5cm x 12.5cm section of the brown spandex cloth material.
3. Cut 4 5cm strips of 5mm foamboard.
4. Using an adhesive material such as super glue, stick the foamboard strips in a square in the centre of the brown spandex cloth material. This is to create a gap between the cloth and the plastic layer to be attached next.
5. Cut a 12.5cm x 12.5cm section of polyethylene plastic.
6. Stick the plastic material onto the spandex cloth using an adhesive material such as super glue.
7. Cut 4 10cm strips of 5mm foamboard.
8. Using an adhesive material such as super glue, stick the 4 foamboard strips around the edges of the plastic material. This is to create sufficient space between the plastic material layer and the skin layer.
9. Cut a 12.5cm x 12.5cm section of artificial skin.
10. Stick the artificial skin layer onto the previously constructed layers using an adhesive material such as super glue.
11. Demarcate 4 incisions by a dotted line.

***Steps to construct box and attachment of hernia sac, Vas deferens, and vessels.***

1. Using a drill or saw, cut a 12.5cm x 12.5cm square in a transparent box with a wooden top.
2. Place a red foam (ethylene-vinyl acetate) with a thickness of 2.6cm inside the transparent box.
3. Cut 3cm of transparent IV tubing and 3cm of blue vessel loops.
4. Attach the IV tubing and vessel loops to the red foam layer using safety pins.
5. Cut a 7cm section of latex balloon with a thickness of 0.75cm.
6. Attach the latex balloon superior to the IV tubing and vessel loops using safety pins.
